# Clinical Value of Bioactive Adrenomedullin and Proenkephalin A in Patients with Left Ventricular Assist Devices: An Observational Study

**DOI:** 10.3390/jcm14103613

**Published:** 2025-05-21

**Authors:** Leyla Dogan, Ahmad Abugameh, Alish Kolashov, Ajay Moza, Andreas Goetzenich, Christian Stoppe, Mohammed Shoaib, Deborah Bergmann, Jan Spillner, Mohammad Amen Khattab, Rashad Zayat

**Affiliations:** 1 Faculty of Medicine, Department of Cardiac Surgery, University Hospital Aachen, RWTH Aachen University, 52074 Aachen, Germany; leyladogan90@web.de (L.D.); amoza@ukaachen.de (A.M.); andreas@goetzenich.net (A.G.); drshoaib80@gmail.com (M.S.); mkhattab@ukaachen.de (M.A.K.); 2 Department of Cardiovascular Surgery, Klinikum Dortmund gGmbH, 44137 Dortmund, Germany; abugameh@online.de; 3 Faculty of Medicine, Witten/Herdecke University, 58453 Witten, Germany; 4 Heart Center Trier, Department of Cardiovascular and Thoracic Surgery, Barmherzige Brueder Hospital, 54292 Trier, Germany; a.kolashov@gmail.com; 5 Medical Affairs, Abiomed GmbH, 52074 Aachen, Germany; 6 Department of Anesthesiology, Intensive Care, Emergency and Pain Medicine, University Hospital Würzburg, 97080 Würzburg, Germany; christian.stoppe@gmail.com; 7 SphingoTec GmbH, 16761 Hennigsdorf, Germany; deborah.bergmann@sphingotec.com; 8 Faculty of Medicine, Department of Thoracic Surgery, University Hospital Aachen, RWTH Aachen University, 52074 Aachen, Germany; jspillner@ukaachen.de

**Keywords:** left ventricular assist devices, right heart failure, proenkephalin A, bioactive adrenomedullin, acute kidney disease

## Abstract

**Background/Objectives**: In the context of acute heart failure, proenkephalin A (penKid) has emerged as a prognostic marker for acute kidney injury (AKI), whereas bioactive adrenomedullin (bio-ADM) has been identified as a significant biomarker linked to shock and organ dysfunction. This raises the question of whether they can serve as predictors of postoperative complications in patients receiving left ventricular assist devices (LVADs). **Methods**: This observational study prospectively enrolled patients who had received LVAD implantation. Routine laboratory values as well as plasma levels of penKid and bio-ADM were assessed at four time intervals, spanning from preinduction of anesthesia to 48 h post surgery. Clinical data, the HeartMate 3-risk-score (HM3RS), HeartMateII-risk-score (HMRS), Michigan-right-heart-failure risk score (MRHFS), Euromacs-RHFS (EURORHFS), and kidney failure risk score (KFR) were calculated. Multivariate logistic regression and receiver operating characteristic (ROC) analysis were performed. We entered the biomarkers with the established risk scores into the models. **Results**: In 20 patients who had undergone LVAD implantation, preoperative penKid level was a predictor of postoperative AKI (OR: 1.05, 95%-CI: 1.0–1.09; *p* = 0.049) and 30-day mortality (OR: 1.01, 95%-CI: 1.0–1.02; *p* = 0.033). Bio-ADM was the only predictor of postoperative right heart failure (RHF) (OR: 1.11, 95%-CI: 1.01–1.23; *p* = 0.034) and rehospitalization (OR: 1.06, 95%-CI: 1.0–1.13; *p* = 0.047). In the ROC analysis, bio-ADM, as a predictor of post-LVAD RHF, had an area under the curve (AUC) of 0.88. When bio-ADM was added to the accepted clinical scores for post-LVAD RHF prediction (CRITT-score, MRHFS, and EURORHFS), the AUC reached 0.98. The AUC for preoperative penKid, as a predictor of postoperative AKI, was 0.95, and after adding its predictive value to the KFR score, the AUC reached 0.97. **Conclusions**: In the present study, the biomarkers penKid and bio-ADM predicted clinically significant patient outcomes after LVAD implantation such as AKI, RHF, and 30-day mortality. Adding biomarkers to well-established risk scores improved the AUC for prediction of postoperative complications.

## 1. Introduction

Various risk stratification models have been proposed to predict survival and postoperative complications such as right heart failure (RHF) and acute kidney injury (AKI) following left ventricular assist device implantation; however, their practical application in real-world decision-making is limited by their reliance on a limited number of variables derived from small datasets or their focus on specific pumps [1,2,3].

The incorporation of biomarkers into existing clinical risk models has the potential to increase the predictive accuracy of unfavorable clinical outcomes.

Proenkephalin A (penKid) is a stable endogenous opioid peptide that has cardiometabolic effects and enhances renal function. PenKid has become a real-time biomarker of AKI since it reacts quickly to kidney malfunction [4,5]. Recent studies have demonstrated that in patients with acute coronary syndrome or heart failure (HF), circulating penKid is a unique indicator of in-hospital AKI and elevated mortality risk [5,6].

Adrenomedullin (bio-ADM) is a protein that plays a significant role in preserving the barrier function of the vascular endothelium [7]. Bio-ADM serves as a potential indicator for residual congestion [8]. It is also significantly correlated with pulmonary capillary wedge pressure and right atrial pressure, while N-terminal pro-B-type natriuretic peptide and increased predischarge levels of Bio-ADM are significantly correlated with HF rehospitalization [7,8,9].

These findings raise the question of whether penKid and bio-ADM can serve as effective biomarkers alongside the established risk scores to predict LVAD outcomes.

## 2. Materials and Methods

### 2.1. Patients

**Figure 1:**
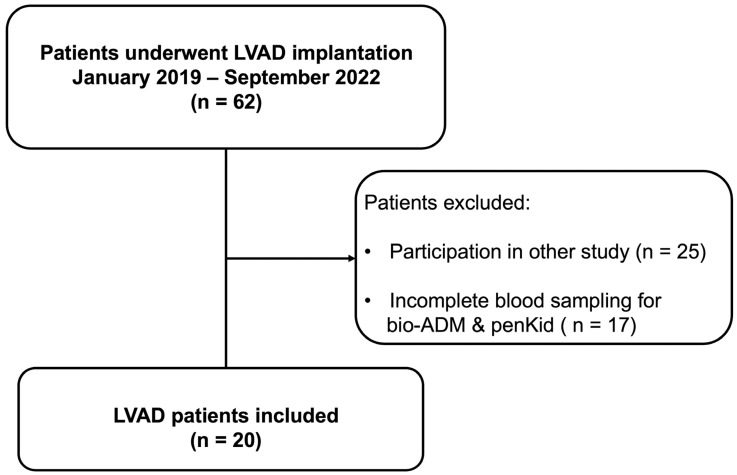
Flowchart.

This prospective observational explorative study conducted at the University Hospital RWTH Aachen (Aachen, Germany) received approval on 10 February 2019 from the institutional review board (Ethics Committee, RWTH Aachen University, Germany), was registered at clinicaltrials.gov (NCT 02488876), and was carried out in accordance with the Declaration of Helsinki. Patients scheduled for elective LVAD implantation were enrolled consecutively from September 2019 until March 2022. Sixty-two patients were screened, and only 20 patients were included (Figure 1 Flowchart). Informed consent was obtained from all enrolled patients before surgery. Exclusion criteria included Interagency Registry for Mechanically Assisted Circulatory Support (Intermacs) level 1, emergency operations, pregnancy, absence of informed consent, or being under 18 years of age.

### 2.2. Surgical Techniques

#### Minimally Invasive Approach

Details about our minimally invasive approach have been described previously [10]. We adopted a modified approach, as described by Schmitto et al. [11]. For the MIS approach, we used bilateral mini-thoracotomy access with a right anterior mini-thoracotomy incision in the second intercostal space and a left lateral thoracotomy through the fifth intercostal space.

### 2.3. Measurement of penKid and bio-ADM

Details of the measurement of both penKid and bio-ADM are described in our previous paper [12]. Blood samples were collected for the measurement of penKid, bio-ADM, and inflammatory markers at several time points: prior to anesthesia induction (S1), upon admission to the intensive care unit (ICU) (S2), and at 24 and 48 h post surgery (S3 and S4). After centrifuging the ethylenediaminetetraacetic acid (EDTA) whole blood samples for 10 min at 3000 rpm, the supernatants were transferred to cryotubes and stored at −80 °C until the analysis had been completed. A reagent (sphingotest^®^ bio-ADM^®^) developed by sphingotecGmbH (Hennigsdorf, Germany) was employed to measure plasma bio-ADM [13]. The bio-ADM immunoassay is a one-step sandwich chemiluminescence immunoassay utilizing acridinium NHS-ester labeling for the detection of human ADM in unprocessed plasma. It employs two mouse monoclonal antibodies: one targeting the mid-region and the other targeting the amidated C-terminal portion of ADM. The test utilizes 50 μL of plasma samples or calibrators and 220 μL of labeled detection antibodies. Bio-ADM in EDTA plasma is stable at room temperature for up to 24 h, and samples can withstand at least four freeze-thaw cycles without degradation. The sensitivity of the analytical test is 2 pg/mL; the lowest detection limit is 3 pg/mL, with intra- and interassay coefficients ranging from 5% to 10% and 4% to 8%, respectively, within the elevated measurement range. This test exhibits great specificity for bio-ADM, reacting exclusively with the mature amidated C-terminus of ADM and not with other forms of (pro-) ADM [13]. In healthy individuals, the median bio-ADM concentration is 20.7 pg/mL (99th percentile: 43 pg/mL) [14].

PenKid was quantified using a sandwich immunoassay (sphingotest^®^ penKid^®^) that was developed by sphingotec GmbH (Hennigsdorf, Germany) and targets PENK A amino acids 119–159 [15]. LD, RZ, and a biologist from our department (SK) received instructions on how to perform the measurements. The measurements performed in our department laboratory by SK, LD, RZ, and DB from the company sphingotecGmbH (Hennigsdorf, Germany) did not include blood sampling or testing for bio-ADM or penKid.

### 2.4. Data Collection

Demographics, clinical course, perioperative data, laboratory data, and postoperative indices, including bleeding, thromboembolic events, RHF, mortality, and AKI, were obtained from our institutional database. Adverse events were defined according to INTERMACS 2021 definition [16]. We calculated the estimated glomerular filtration rate (eGFR) according to the National Kidney Foundation and the American Society of Nephrology algorithms [17]. The kidney failure risk equation (KFR) was calculated [18].

We also calculated known established risk scores for the prediction of outcomes after LVAD implantation: the HeartMate II risk score (HMRS) [2], the HeartMate 3 risk score (HM3RS) [19], the European system for cardiac operative risk evaluation II (EuroSCORE II) [20], the Michigan RHF score (MRHFS) [21], the CRITT RHF score (CRITT) [22], and the European Registry for Patients with Mechanical Circulatory Support RHF score (EURORHFS) [23].

### 2.5. Statistical Analysis

Categorical variables are displayed as absolute numbers and percentages. The chi-square test or Fisher’s exact test was used to analyze categorical variables. The Kolmogorov-Smirnov test was used to assess the normality of the data. Normally distributed continuous variables are expressed as means ± standard deviations. Nonnormally distributed variables are expressed as medians accompanied by interquartile ranges (IQRs). We performed a Spearman’s correlation analysis to detect whether there was a correlation between the bio-ADM values and penKid values measured at different time points with the postoperative outcomes. A logistic regression analysis was conducted to ascertain whether bio-ADM and penKid could predict postoperative outcomes such as mortality, RHF, sepsis, AKI with the need for postoperative dialysis, and rehospitalization due to HF decompensation. In the next step, we performed multivariable logistic regression and added bio-ADM, penKid, and all other established risk scores to detect which parameters remained predictors of postoperative outcomes. As a third step, we performed receiver operating characteristic (ROC) analysis to calculate the area under the curve (AUC) for all risk scores and biomarkers separately. Then, we added the predicted value of bio-ADM to the scores that predict adverse events and scores that predict RHF. The predictive value of penKid was tested separately in the ROC analysis, and its value was added to the scores that predict mortality and to the KFR score. Delong’s test was used to compare the differences between the AUCs. We employed the usual breps(#) in Stata/IC version 16.1, which utilizes the number of bootstrap replications; the default setting is breps(1000) for the rocreg command. We employed the following bootstrap method in Stata for the logistic regression: (bootstrap stat = r(mystat), reps(100): myprog1 version 1). For cross-validation, we utilized the crossvalidate2 tool in Stata and selected a 60/20/20 split with four folds to assess the accuracy of the logistic regression model. Additionally, we conducted a post-estimation analysis to compute the adjusted *p*-values in the multivariate regression analysis.

All *p* values were two-tailed, with a significance threshold set at <0.05. All analyses were performed via STATA (StataCorp. 2019. Stata Statistical Software: Release 16. StataCorp LLC: College Station, TX, USA) and with the jamovi project (2020) (jamovi software, version 2.6.3; JAMovi.org), which is accessible at https://www.jamovi.org/ (accessed on 15 January 2025). GraphPad Prism version 10.4.6 for Mac OS X, GraphPad Software, Boston, MA, USA, www.graphpad.com (accessed on 18 January 2025), was used to visualize some of the results.

## 3. Results

### 3.1. Participants

Details on patient demographics, preoperative right heart catheterization results, and preoperative laboratory results are presented in Table 1. Between January 2019 and September 2022, 62 LVADs were implanted in our department. Twenty patients were included in the study (Figure 1 shows the flowchart). After they had consented to participate, we were able to collect all needed blood samples. Among the 20 included patients, the mean age was 65.25 ± 10.68 years, and 25% were female (Table 1). All patients received a HeartMate 3 (HM3; Abbott, Abbott Park, IL, USA) LVAD. Thirty-five percent and 55% of patients were at INTERMACS level 3 and 4, respectively. Twenty-five percent of patients had preoperative CKD, and no patient had preoperative dialysis. Seventy-five percent of the patients had pulmonary hypertension. According to the echocardiographic parameters RV fractional area change (RVFAC: 35.86 ± 10.36%) and tricuspid annular systolic velocity (TASV: 12.00 ± 14.22 cm/s), none of the patients had severe or moderate RV dysfunction preoperatively (Table 1).

The mean HM3RS score for all patients was 3.37 ± 1.43, the mean HMRS score was 1.33 ± 0.80, the mean EuroSCORE II score was 11.86 ± 6.79%, the mean MRHFS score was 1.23 ± 1.63, the mean CRITT score was 2.98 ± 2.20, the mean EURORHFS score was 2.98 ± 2.20, and the mean KFR score was 3.22 ± 6.95 (Table 1). Perioperative data are presented in Table 1.

Table 2 includes details about postoperative complications. Twenty percent of the patients developed septic shock, 50% suffered postoperative AKI, 35% required temporary dialysis, 30% had RHF, and 10% required the implantation of a temporary RVAD system (CentriMag, Levitronix LLC, Waltham, MA, USA).

### 3.2. Time Course of penKid and bio-ADM

**Figure 2:**
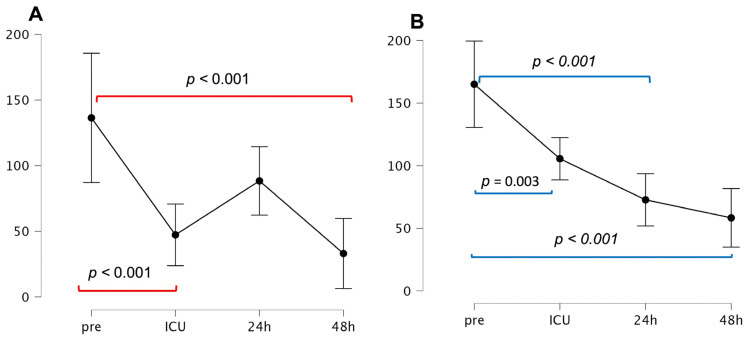
Time course of bio-ADM and penKid. (**A**): Time course of bioactive adrenomedullin; (**B**): time course of penKid A, ICU: intensive care unit; preop: preoperative; postop: postoperative.

Figure 2 shows the time course of bio-ADM and penKid. As expected, the preoperative plasma bio-ADM level was 52 (39, 247) pg/mL, which is higher than that in the healthy population, i.e., 44.1 (25.9, 82.7) pg/mL [24]. After LVAD implantation, there was a significant decrease in the plasma bio-ADM levels compared with the preoperative values (preoperative vs. ICU admission: 52 (39, 247) pg/mL vs. 19.8 (15.6, 76) pg/mL, *p* < 0.001), which increased, but not statistically significantly, at 24 h post surgery to 36.4 (20, 137) pg/mL (*p* = 0.107) (Figure 1) and decreased again, this time significantly, 48 h post-LVAD implantation compared to the preoperative value, i.e., 52 (39, 247) vs. 16.6 (12, 40.7) pg/mL, *p* < 0.001.

Similarly, the median preoperative plasma penKid level was significantly greater, at 131.36 (82.18, 198.0) pmol/L, than the normal value in the healthy population, i.e., 50.22 (43.54, 58.52) pmol/L [25].

As soon as the LVAD had been implanted and the heart was unloaded and recompensated, the plasma bio-ADM and penKid levels decreased significantly (penKid: preoperative vs. ICU admission: 131.36 (82.18, 198.0) pmol/L vs. 63.37 (55.91, 165.25) pmol/L, *p* < 0.001). The plasma penkid concentration continued to decrease significantly compared with the preoperative value (preoperative vs. 48 h postoperative: 131.36 (82.18, 198.0) pmol/L vs. 49.4 (40.0, 59.6) pmol/L, *p* < 0.00) (Figure 2).

### 3.3. Preoperative Plasma bio-ADM Predicts Postoperative RHF and Hospital Readmission Due to HF

Preoperative bio-ADM showed very good correlation with post-LVAD RHF (*r* = 0.80, *p* < 0.001), and bio-ADM at ICU showed good correlation with post-LVAD RHF (*r* = 0.65, *p* < 0.001); the same was found for bio-ADM at 24 h and at 48 h postoperatively (*r* = 0.80, *p* < 0.001 and *r* = 0.62, *p* = 0.004). However, in the binominal multivariate regression analysis, only the preoperative bio-ADM remained a significant predictor for post-LVAD RHF when entered with the established risk scores (MRHFS, CRITT score, and EURORHFS; see Supplement File S1). The same analysis was applied to the bio-ADM values at different time points to detect the correlation with rehospitalization (see Supplement File S1).

After logistic regression with RHF as the dependent variable and considering the preoperative bio-ADM value, MRHFS, EURORHFS, and CRITT score, only preoperative bio-ADM remained a significant independent predictor of postoperative RHF after LVAD implantation (OR: 1.01 (95%-CI: 0.99–1.03), *p* = 0.034; see Supplementary Table S1). After entering the preoperative bio-ADM in a multivariate logistic regression with EuroSCOREII, HM3RS, and HMRS with rehospitalization as the dependent variable and adjusting for multiple variables, none of the variables remained as an independent predictor for rehospitalization (Supplementary Table S1).

**Figure 3:**
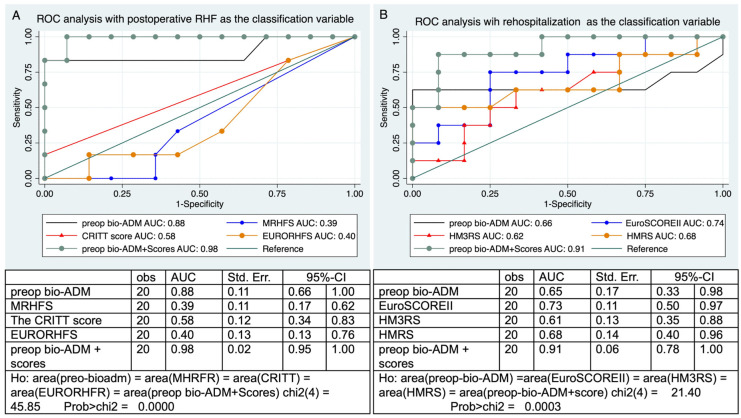
ROC analysis of bio-ADM and other risk scores for the prediction of postoperative right heart failure and rehospitalization due to heart failure. AUC: are under the curve: Bio-ADM: bioactive adrenomedullin; CI: confidence interval; EURORHFS: EUROMACS right heart failure risk score; EuroSCORE II: European system for cardiac operative risk evaluation II; HMRS: the HeartMate II risk score; HM3RS: the HeartMate 3 risk score; MRHFS: the Michigan RHF score; preop: preoperative; St. Err.: standard error. (**A**) ROC analysis for prediction of RHF post-LVAD. (**B**) ROC analysis for prediction of rehospitalization after LVAD implantation.

In the next step, we performed ROC analysis and calculated the AUC of preoperative bio-ADM with the other risk scores for the prediction of postoperative RHF. Then, we added the prediction value of the bio-ADM to the established known scores. The AUC of preoperative bio-ADM as a predictor of RHF was 0.88, with a sensitivity of 83% and specificity of 100%. The AUC of MRHFS was 0.39, the AUC of the CRITT score was 0.58, and the AUC of the EURORHFS was 0.40. After adding the predictive value of bio-ADM to the other risk scores, the AUC reached 0.98, with a sensitivity of 100% and specificity of 92% (Figure 3A). Additionally, a significant difference between the ROC curves was revealed by Delong’s test *(p* < 0.0001).

The same method was used to calculate the AUC for preoperative bio-ADM, HMRS, HM3RS, and EuroSCORE II using rehospitalization due to HF as the dependent variable. The AUC for the EuroSCOREII was 0.73, the AUC for the HM3RS was 0.61, the AUC for the HMRS was 0.68, the AUC for preoperative bio-ADM was 0.65, and the AUC for preoperative bio-ADM + scores was 0.91, with a sensitivity of 87% and specificity of 91%. Delong’s test also demonstrated a significant disparity between the ROC curves (*p* = 0.0003; see Figure 3B).

### 3.4. Preoperative Plasma penKid Predicts Postoperative AKI with the Need for Dialysis, 30-Day Mortality, and Postoperative Sepsis After LVAD Implantation

In the multivariable logistic regression using the postoperative need for dialysis, we entered the preoperative penKid value and the KFR equation, and only the preoperative penKid remained a significant predictor of postoperative AKI with the need for dialysis after adjustment for multiple variables (OR: 1.05 (95%-CI: 1.00–1.08), *p* = 0.030) (Supplementary Table S2). We then used postoperative sepsis as the dependent variable in a multivariable logistic regression and entered the preoperative penKid value, EuroSCORE II, HM3RS, and HMRS. However, only preoperative penKid remained a significant predictor of postoperative sepsis (OR: 1.01 (95%-CI: 1.00–1.03), *p* = 0.041) (Table S2). We performed further multivariable logistic regression using 30-day mortality as the dependent variable and entered the preoperative penKid value, EuroSCORE II, HM3RS, and HMRS. Only preoperative penKid remained a significant predictor of 30-day mortality (OR: 1.02 (95%-CI: 1.00–1.02), *p* = 0.041; see Table S2).

In the correlation analysis (Supplementary File S2), the preoperative penKid value showed a very good correlation with post-LVAD need of dialysis (*r* = 0.78, *p* < 0.001) and penKid at ICU showed a correlation with post-LVAD dialysis (*r* = 0.58, *p* = 0.008); the same was found for penKid at 24 h post surgery (*r* = 0.57, *p* = 0.009). However, in the binomial multivariate regression analysis, the preoperative penKid the penKid value at ICU admission showed a prediction value after it was entered with the KFS (OR: 1.08, 95%-CI: 0.76–1.55, *p* = 0.027; see Supplementary File S2).

The same analysis was applied for penKid values at different time points to detect the correlation with 30-day mortality. Besides the preoperative penKid values, penKid values at ICU admission showed a weak correlation with 30-day mortality (*r* = 0.55, *p* = 0.013). After entering penKid values at ICU admission with the established scores (EuroSCOREII, HMRS, HM3RS), penKid at ICU admission was found to have no predictive value for 30-day mortality (Supplement File S2).

**Figure 4:**
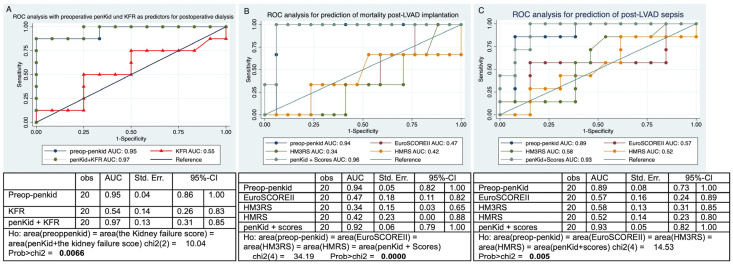
ROC curve analysis of penKid and other risk scores for the prediction of 30-day mortality, postoperative sepsis, and postoperative dialysis. AUC: area under the curve; 95%-CI: 95% confidence interval; penKid: proenkephalin A; EuroSCORE II: European system for cardiac operative risk evaluation II; HMRS: the HeartMate II risk score; HM3RS: the HeartMate 3 risk score; preop: preoperative; KFR: kidney failure risk equation; St. Err.: standard error.

In the ROC analysis for the prediction of postoperative 30-day mortality, the AUC for preoperative penKid was 0.94, that for the EuroSCORE II was 0.47, that for the HM3RS was 0.34, that for the HMRS was 0.42, and that for the preoperative penKid+ risk scores was 0.92, with a sensitivity of 100% and specificity of 88% (Figure 4A). Delong’s test revealed a significant difference between the ROC curves (*p* < 0.0001). Although we performed ROC analysis for the prediction of postoperative sepsis, the AUC for the preoperative penKid was 0.89, that for the EuroSCORE II AUC was 0.57, that for the HM3RS was 0.58, and that for the HMRS was 0.52. After adding the predictive value of the preoperative penKid to the risk scores, the AUC reached 0.93, with a sensitivity of 100% and specificity of 84% (Figure 4B). Additionally, a substantial difference between the ROC curves was revealed by Delong’s test (*p* = 0.005). ROC analysis for the prediction of postoperative dialysis revealed that the AUC for preoperative penKid was 0.95, that for the KFR was 0.54, and that for preoperative penKid + KFR reached 0.97, with a sensitivity of 87% and specificity of 100% (Figure 4C). Delong’s test indicated a significant disparity in the ROC curves, i.e., *p* = 0.0066.

## 4. Discussion

This observational study of LVAD patients demonstrated that bio-ADM is an effective biomarker for the detection of postoperative RHF, as well as for assessing the risk of rehospitalization due to HF decompensation. Conversely, penKid proved to be a reliable biomarker for identifying the occurrence of postoperative AKI with the need for dialysis and for predicting both 30-day mortality and postoperative sepsis. This is, to our knowledge, the first presentation of data concerning penKid and bio-ADM utilized concurrently in patients receiving LVADs. Bio-ADM added a predictive value to establish scores that predict post-LVAD RHF (EURORHFS, MRHFS, the CRITT score) and to scores that predict worse outcomes after LVAD implantation (HM3RS, HMRS, EuroSCORE II). On the other hand, penKid provided a predictive benefit in adding predictive value to the KFR regarding postoperative AKI and added a predictive value for scores that predict survival after LVAD implantation (HM3RS, HMRS, EuroSCORE II).

Our data revealed that the circulating levels of penKid decreased over time, whereas the opposite was observed for the plasma values of bio-ADM, which increased after LVAD implantation. The time courses of both penKid and bio-ADM reflect the early effect of LVAD implantation, which leads to unloading and recompensation of the heart.

Bio-ADM performs numerous activities inside humans, such as regulating blood pressure, maintaining endothelial barrier integrity, and modulating immune responses, while also exhibiting cardioprotective properties, as described in a recent review by Geven et al. [26].

The prognostic significance of bio-ADM has been demonstrated in many critical circumstances [7,8,12,25]. Furthermore, its predictive function has recently been expanded to the HF patient context. Our results are supported by those of Self et al. [24], who demonstrated that in patients with acute HF (AHF), elevated bio-ADM levels correlated with the composite primary outcome, which included mortality, rehospitalization, emergency dialysis, cardiac arrest with resuscitation, respiratory failure, extended hospitalization, and acute coronary syndrome within 30 days. Our findings regarding bio-ADM and penKid are also in accordance with the results from a study by Molvin et al. [27], who measured bio-ADM and penKid in 530 subjects hospitalized for AHF. They reported that penKid was an independent predictor of AKI and demonstrated that both penKid and bio-ADM were associated with in-hospital mortality and one-year mortality [27].

Despite advancements in technology and surgical expertise, approximately 15% of patients with LVAD experience RHF [28]. Currently, a minimum of six risk scores designed to predict RHF following LVAD implantation have been established [29]. The assessment of a candidate’s risk for RHF incorporates various parameters, including clinical indicators (such as the need for inotropic support, vasopressors, ventilators, and/or intra-aortic balloon pump support), laboratory values (serum creatinine, bilirubin, and hepatic transaminases), hemodynamic measurements (right atrial pressure and the RA/wedge pressure ratio and RV stroke work index), and echocardiographic findings (severe tricuspid regurgitation and severe RV dysfunction). Nonetheless, risk discrimination among the various scores remains inadequate, and no individual risk score or echocardiographic parameter provides the necessary sensitivity or specificity to reliably predict the requirement of biventricular assist device support in the pre-VAD context.

In our study, we showed that adding the bio-ADM biomarker to established risk scores for predicting RHF (CRITT score, MRHFS, EURORHFS) improved the AUC and increased the sensitivity and specificity.

PenKid serves as an inflammation-independent indicator of renal function, facilitating the early detection of AKI by predicting subsequent alterations in serum creatinine levels [12,30]. The highly dynamic nature of penKid facilitates close monitoring of renal function. Our study revealed penKid to be an independent predictor of postoperative AKI and the need for dialysis, a predictor of postoperative sepsis, and an independent predictor of 30-day mortality after LVAD implantation. Our results align with the findings of Molvin et al. [27] and the study by Ng et al. [6] in the context of AHF. The proposed pathophysiological mechanism for pneumonia in the context of AKI and its prognostic significance for mortality may be associated with the cardio-depressive effects of enkephalin [31], leading to diminished kidney perfusion and the progression of heart failure [31]. Findings from the Acute Decompensated Heart Failure National Registry indicate that the administration of opiates is associated with a poorer prognosis in heart failure patients [32].

Bio-ADM and penKid likely represent distinct pathophysiological mechanisms (i.e., bio-ADM associated with congestion and post-LVAD RHF and penKid associated with post-LVAD AKI). However, their combination may provide complementary insights into cardiorenal syndrome in patients with AHF. Additional prospective studies are necessary. For example, in a clinical setting, bio-ADM and penKid may serve as valuable tools for guiding diuretic therapy. Increased bio-ADM correlates with clinical congestion [8], suggesting its potential utility in identifying individuals requiring intensified diuretic therapy. The treatment of AHF and decongestion may pose risks to renal function. The integration of these two biomarkers is beneficial, as penKid can assist in directing treatment to preserve kidney function.

Many risk scores exist for predicting adverse outcomes after cardiac surgery and after LVAD implantation. These models may exhibit variable performance across patients owing to the heterogeneity inherent in this population and the procedures involved. The European Association for Cardio-Thoracic Surgery (EACTS) indicates that current risk models are poorly calibrated, especially in high-risk patients, highlighting the necessity for improvements in risk prediction quality [33]. INTERMACS classifications give granularity for identifying and risk stratifying advanced HF patients, although they have limits. These include a lack of patient feature input, clinical variability/sub-classifications within a profile, fluctuating levels during a patient’s pre-implant course, and subjective assessment by the person reporting implant characteristics to INTERMACS.

The incorporation of biomarkers into established risk scores may enhance their predictive values. An earlier effort to incorporate a panel of biomarkers (ST2, galectin-3, N-terminal pro-brain natriuretic peptide, cystatin C, and interleukins 6 and 10) into a well-established risk model, i.e., that of The Society of Thoracic Surgeons (STS), demonstrated an enhancement in the AUC/ROC from 0.66 to 0.74 (*p =* 0.0001) within the derivation cohort; however, external validation yielded suboptimal results (AUC/ROC 0.51) [34].

Our study demonstrated that incorporating biomarkers into these risk scores enhances their predictive value. Although bio-ADM and penKid likely represent distinct pathophysiological mechanisms (i.e., bio-ADM is associated with congestion and penKid with worsening renal function), their combination may provide complementary insights into cardiorenal syndrome in patients with acute heart failure.

### Strengths and Limitations of the Study

The merits of our study include (a) the standardized evaluation of adverse events by non-research personnel (seasoned intensive care physicians and cardiothoracic surgeons), (b) the external assessment of biomarkers by individuals uninformed of the patient’s clinical trajectory, and (c) the emphasis on preoperative, intraoperative, and postoperative biomarker values.

We acknowledge several limitations of our study. The small sample size restricted generalizability, rendering our results solely as the basis for hypotheses. The sample size precluded any definitive findings on high-risk individuals owing to the intricacies of surgery and comorbidities. The small sample size may have led to both type I and type II errors, which may have resulted in erroneous findings, squandered resources, or possibly detrimental judgements. Nonetheless, the measurement of both Bio-ADM and penKid in LVAD patients pre- and postoperatively has been infrequently documented in the literature, and the high AUC identified in our research, together with the very narrow 95% confidence interval for the odds ratio, reinforces the validity of the odds ratio. The diagnostic and prognostic efficacy of bio-ADM and penKid requires validation through prospective studies including a large number of LVAD patients. Finally, our findings cannot be extrapolated to chronic HF patients, nor are they applicable to those with moderate decompensated HF, because we included only patients with end-stage HF requiring LVAD implantation.

## 5. Conclusions

In the present study, bio-ADM and penKid demonstrated encouraging results as a tool for forecasting the prediction of post-LVAD RHF, postoperative AKI with the need for dialysis, 30-day mortality, and sepsis. Additionally, adding the predictive value of bio-ADM and penKid to the appropriate established risk scores provided a higher AUC and superior predictive value. A concurrent assessment of these two biomarkers in patients with LVAD may yield critical insights for therapeutic decision-making aimed at decreasing mortality, AKI, and readmission rates. However, due to the small sample size, further prospective multicentre studies including a large number of LVAD patients are necessary to validate the diagnostic and prognostic value of bio-ADM and penKid.

## Figures and Tables

**Table 1:** Preoperative patients characteristics, echocardiographic parameter, laboratory, right heart catheterization, and perioperative data.

Age years	65.25 ± 10.68
Female n (%)	5 (25.0)
ICM n (%)	13 (65.0)
DCM n (%)	7 (35.0)
IDDM n (%)	4 (20.0)
PAD n (%)	3 (15.0)
CVD n (%)	1 (5.0)
AHT n (%)	13 (65.0)
Nicotine n (%)	11 (55.0)
Preoperative Apoplex n (%)	2 (10.0)
HLP n (%)	6 (30.0)
COPD n (%)	4 (20.0)
PHT n (%)	15 (75.0)
Prior cardiac surgery n (%)	2 (10.0)
INTERMACS 2 n (%)	2 (10.0)
INTERMACS 3 n (%)	7 (35.0)
INTERMACS 4 n (%)	11 (55.0)
BMI kg/m^2^	29.05 ± 6.06
BSA m^2^	2.09 ± 0.30
EF %	20.40 ± 4.52
EuroSCORE II %	11.86 ± 6.79
HM3RS	3.37 ± 1.43
HMRS	1.33 ± 0.80
MRHFS	1.23 ± 1.63
CRITT score	0.95 ± 0.69
EURORHFS	2.98 ± 2.20
The kidney failure equations	3.22 ± 6.95
CKD	5 (25%)
LVEDD cm	6.90 ± 1.53
RVFAC %	35.86 ± 10.36
TASV cm/s	12.00 ± 14.22
TAPSE cm	9.57 ± 6.23
Sphericity index	0.74 ± 0.15
**Preoperative laboratory:**	
Creatinine mg/dL	1.35 ± 0.65
eGFR mL/min/1.73 m^2^	68.77 ± 18.36
BUN mg/dL	50 (39, 57.50)
Albumin g/dL	3.46 ± 0.75
INR	1.12 ± 0.26
Thrombocytes/nL	220 (179.25, 268.75)
AST U/L	29.50 (22, 34.50)
Hematocrit %	40.12 ± 5.78
Bilirubin mg/dL	0.72 ± 0.49
**Preoperative right heart catheterization:**	
CVP mmHg	12.45 ± 6.39
RV pressure mmHg	46.21 ± 13.79
mPAP mmHg	35.40 ± 16.28
sPAP mmHg	48.19 ± 16.20
PCWP mmHg	23.25 ± 9.74
RA/PCWP	0.63 ± 0.38
**Perioperative data:**	
CPB time minute	129.30 ± 62.68
Cross clamp time	3.60 ± 12.84

AHT: arterial hypertension; AST: aspartate aminotransferase; BMI: body mass index Kg/m^2^; BSA: body surface area m^2^; BUN: blood urea nitrogen; CPB: cardiopulmonary bypass; CKD: chronic kidney disease; COPD: chronic obstructive pulmonary disease; CVD: cerebrovascular disease; CVP: central venous pressure; DCM: dilative cardiomyopathy; EF: ejection fraction; EURORHFS: The European Registry for Patients with Mechanical Circulatory Support right heart failure score; EuroSCORE II: European system for cardiac operative risk evaluation II; HMRS: the HeartMate II risk score; HM3RS: the HeartMate 3 risk score; IDDM: insulin-dependent diabetes mellitus; HLP: hyperlipoproteinemia; INR: international normalized ratio; ICM: ischemic cardiomyopathy; INTERMACS: Interagency Registry for Mechanically Assisted Circulatory Support; mPAP: mean pulmonary artery pressure; MRHFS: the Michigan RHF score; PAD: peripheral arterial disease; PHT: pulmonary hypertension; RA/PCWP: right atrium pressure/postcapillary wedge pressure ratio; RVFAC: right ventricle fractional area ratio; TASV: tricuspid annular systolic velocity; TAPSE: tricuspid annular plane systolic excursion; RV: right ventricle; sPAP: systolic pulmonary pressure; PCWP: postcapillary wedge pressure.

**Table 2:** Postoperative complications.

ICU days	8.38 (4, 23.69)
Hospital lOS days	22 (16, 33)
Inotropic Days	7.88 (2.75, 18.25)
iNO Hours	15.50 (0, 69.25)
ICU readmission n (%)	3 (15.0)
Postop Pneumonia n (%)	14 (70.0)
Postop Sepsis n (%)	4 (20.0)
Delirium n (%)	4 (20.0)
AKI n (%)	10 (50.0)
Postop dialysis n (%)	8 (40.0)
re-thorax n (%)	7 (35.0)
Right-Heart-Failure n (%)	6 (30.0)
RVAD Implantation n (%)	2 (10.0)
Device thrombus n (%)	1 (5.0)
Ischemic stroke n (%)	1 (5.0)
Hemorrhagic stroke n (%)	1(5.0)
TIA n (%)	1(5.0)
30 days mortality	3 (15)
Overall mortality within 2 years	3 (15)

AKI: Acute kidney injury; ICU: Intensive care unit; iNO: inhaled nitroxide; LOS: length of stay; RVAD: Right ventricular assist device; TIA: transitory ischemic attack.

## Data Availability

The data that support the findings of this study are available on request from the corresponding author. The data are not publicly available due to privacy or ethical restrictions.

## Supplementary Materials

The following supporting information can be downloaded at: https://www.mdpi.com/article/10.3390/jcm14103613/s1, Table S1: Multivariate logistic regression with post-LVAD RHF as a dependent variable. Table S2: Multivariate logistic regression with post-LVAD dialysis as a dependent variable. File S1: Correlation matrix between bio-ADM at different time points and post-LVAD RHF. File S2: Correlation analysis between penKid measurements at different time points and postoperative dialysis.

## Abbreviations

The following abbreviations are used in this manuscript:

95%-CI	95% confidence interval
AHT	Arterial hypertension
AST	Aspartate aminotransferase
AKI	Acute kidney injury
AUC	area under the curve
BMI	Body mass index Kg/m^2^
BSA	Body surface area m^2^
BUN	Blood urea nitrogen
Bio-ADM	Bioactive adrenomedullin
CPB	Cardiopulmonary bypass
CKD	Chronic kidney disease
COPD	Chronic obstructive pulmonary disease
CRITT score	CVP greater than 15 mm Hg (C); severe RV dysfunction (R); preoperative mechanical ventilation/intubation (I); severe tricuspid regurgitation (T); and tachycardia (T)
CVD	Cerebrovascular disease
CVP	Central venous pressure
DCM	Dilative cardiomyopathy
EDTA	ethylenediaminetetraacetic acid blood sample
eGFR	estimated glomerular filtration rate
EuroSCORE II	European system for cardiac operative risk evaluation II
EURORHFS	The European Registry for Patients with Mechanical Circulatory Support right heart failure score
IDDM	Insulin-dependent diabetes mellitus
HLP	Hyperlipoproteinemia
HMII	HeartMate II
HM3	HeartMate 3
HMRS	The HeartMate II-risk-score
HM3RS	The HeartMate 3-risk-score
HF	Heart failure
ICU	Intensive care unit
iNO	Inhaled nitroxide
INTERMACS	the Interagency Registry for Mechanically Assisted Circulatory Support
INR	International normalized ratio
ICM	Ischemic cardiomyopathy
KFR	kidney failure risk score
LVADs	Left ventricular assist devices
LOS	Length of stay
MRHFS	Michigan-right-heart-failure risk score
mPAP	mean pulmonary artery pressure
OR	Odds ratio
PAD	Peripheral arterial disease
Penkid	Proenkephalin A
PHT	Pulmonary hypertension
RAP/PCWP	right atrium pressure/postcapillary wedge pressure ratio
ROC	Receiver operating characteristic analysis
RHF	Right heart failure
RVAD	Right ventricular assist device
RVFAC	RV fractional area change
sPAP	Systolic pulmonary pressure
TASV	Tricuspid annular systolic velocity
TAPSE	Tricuspid annular plane systolic excursion
TIA	Transitory ischemic attack
