# Supplementary material for: Clinical Value of Bioactive Adrenomedullin and Proenkephalin A in Patients with Left Ventricular Assist Devices: An Observational Study
Source: J Clin Med. 2025 May 21;14(10):3613. doi: 10.3390/jcm14103613 (PMC12112301; doi:10.3390/jcm14103613)
Supplement: Supplementary file 1 [file jcm-14-03613-s001.zip › Supplementary File S1.pdf]

Supplementary File S1

Correlation matrix between bio-ADM at different time points and post-LVAD RHF

Correlation Matrix

Correlation Matrix

Correlation Matrix

		postoop_RHF	pre Bio-ADM	bio-ADM at ICU	Bio-ADM 24h	Bio-ADM 48h
postoop_RHF	Spearman's rho	—				
	df	—				
	p-value	—				
	N	—				
pre Bio-ADM	Spearman's rho	0.80***	—			
	df	18	—			
	p-value	<.001	—			
	N	20	—			
bio-ADM at ICU	Spearman's rho	0.65***	0.79***	—		
	df	18	18	—		
	p-value	<.001	<.001	—		
	N	20	20	—		
Bio-ADM 24h	Spearman's rho	0.80***	0.90***	0.90***	—	
	df	18	18	18	—	
	p-value	<.001	<.001	<.001	—	
	N	20	20	20	—	
Bio-ADM 48h	Spearman's rho	0.62**	0.37	0.59**	0.43	—
	df	18	18	18	18	—
	p-value	0.004	0.106	0.006	0.061	—
	N	20	20	20	20	—

Note. * p < .05, ** p < .01, *** p < .001

Binomial Logistic Regression using bio-ADM at ICU admission post-LVAD with other risk scores to predict post-LVAD RHF

Model Fit Measures

Model	Deviance	AIC	R^2_{McF}
1	0.00	10.00	1.00

Note. Models estimated using sample size of N=19

Model Coefficients - postoop_RHF

						95% Confidence Interval	
Predictor	Estimate	SE	Z	p	Odds ratio	Lower	Upper
Intercept	-41.29	129002.88	-0.00	1.000	0.00	0.00	Inf
Michigan_RV	-1.59	108957.05	-0.00	1.000	0.20	0.00	Inf
CRITT	7.67	80749.76	0.00	1.000	2147.95	0.00	Inf
EUROMACS-RHF-Score	-3.84	61405.66	-0.00	1.000	0.02	0.00	Inf
bio-ADM at ICU	0.80	1960.43	0.00	1.000	2.21	0.00	Inf

Note. Estimates represent the log odds of "postoop_RHF = 1" vs. "postoop_RHF = 0"

Binomial Logistic Regression using bio-ADM 24h post-LVAD with other risk scores to predict post-LVAD RHF

Model Fit Measures

Model	Deviance	AIC	R^2_{McF}
1	0.00	10.00	1.00

Note. Models estimated using sample size of N=19

Model Coefficients - postoop_RHF

Predictor	Estimate	SE	Z	p	Odds ratio	95% Confidence Interval	
						Lower	Upper
Intercept	-38.28	155784.85	-0.00	1.000	0.00	0.00	Inf
Michigan_RV	-5.68	66971.68	-0.00	1.000	0.00	0.00	Inf
CRITT	5.26	193766.84	0.00	1.000	192.93	0.00	Inf
EUROMACS-RHF-Score	-2.99	35339.61	-0.00	1.000	0.05	0.00	Inf
Bio-ADM 24h	0.53	1136.31	0.00	1.000	1.69	0.00	Inf

Note. Estimates represent the log odds of "postoop_RHF = 1" vs. "postoop_RHF = 0"

Binomial Logistic Regression using bio-ADM 48h post-LVAD with other risk scores to predict post-LVAD RHF

Model Fit Measures

Model	Deviance	AIC	R^2_{McF}
1	0.00	10.00	1.00

Note. Models estimated using sample size of N=19

Model Coefficients - postoop_RHF

Predictor	Estimate	SE	Z	p	Odds ratio	95% Confidence Interval	
						Lower	Upper
Intercept	-389.07	257752.6	-0.00	0.999	0.00	0.00	Inf
Bio-ADM 48h	6.10	3992.78	0.00	0.999	446.97	0.00	Inf

Michigan_RV	-36.79	41125.24	-0.00	0.999	0.00	0.00	Inf
CRITT	168.52	112283.84	0.00	0.999	1.5436149850273904e+73	0.00	Inf
EUROMACS-RHF-Score	-26.01	27785.76	-0.00	0.999	0.00	0.00	Inf

Note. Estimates represent the log odds of "postoop_RHF = 1" vs. "postoop_RHF = 0"

Correlation matrix between bio-ADM at different time points and rehospitalisation

Correlation Matrix

Correlation Matrix

Correlation Matrix

		rehospitalization	Pre bio-ADM	bio-ADM at ICU	Bio-ADM 24h	Bio-ADM 48h
**rehospitalization**	**Spearman's rho**	—				
	**df**	—				
	**p-value**	—				
	**N**	—				
**Pre bio-AD**	**Spearman's rho**	0.78***	—			
	**df**	18	—			
	**p-value**	0.004	—			
	**N**	20	—			
**bio-ADM at ICU**	**Spearman's rho**	0.77***	0.79***	—		
	**df**	18	18	—		
	**p-value**	<.001	<.001	—		
	**N**	20	20	—		
**Bio-ADM 24h**	**Spearman's rho**	0.68***	0.90***	0.90***	—	
	**df**	18	18	18	—	
	**p-value**	<.001	<.001	<.001	—	

	**N**	20	20	20	—	
**Bio-ADM 48h**	**Spearman's rho**	0.27	0.37	0.59**	0.43	—
	**df**	18	18	18	18	—
	**p-value**	0.257	0.106	0.006	0.061	—
	**N**	20	20	20	20	—

Note. * p < .05, ** p < .01, *** p < .001

Binomial Logistic Regression using Bio-ADM at ICU admission and established risk-score to predict rehospitalization

Binomial Logistic Regression

Model Fit Measures

Model	Deviance	AIC	R^2_{McF}
1	9.39	19.39	0.65

Note. Models estimated using sample size of N=20

Model Coefficients - rehospitalization

						95% Confidence Interval	
**Predictor**	**Estimate**	**SE**	**Z**	**p**	**Odds ratio**	**Lower**	**Upper**
Intercept	-5.03	4.35	-1.16	0.247	0.01	0.00	32.84
bio-ADM at ICU	0.15	0.11	1.37	0.170	1.16	0.94	1.42
EuroSCOREII	-0.02	0.15	-0.15	0.885	0.98	0.74	1.30
HeartMate3 risk score	-0.18	1.38	-0.13	0.895	0.83	0.06	12.37
the HMII risk score	0.45	1.48	0.30	0.761	1.57	0.09	28.50

Note. Estimates represent the log odds of "rehospitalization = 1" vs. "rehospitalization = 0"

Binomial Logistic Regression using Bio-ADM at 24h postoperatively and established risk-score to predict rehospitalization

Model Fit Measures

Model	Deviance	AIC	R^2_{McF}
1	11.28	21.28	0.58

Note. Models estimated using sample size of N=20

Model Coefficients - rehospitalization

Predictor	Estimate	SE	Z	p	Odds ratio	95% Confidence Interval	
						Lower	Upper
Intercept	-5.06	4.57	-1.11	0.268	0.01	0.00	49.46
EuroSCOREII	-0.02	0.11	-0.20	0.844	0.98	0.79	1.21
HeartMate3 risk score	0.29	1.16	0.25	0.801	1.34	0.14	12.87
the HMII risk score	0.48	1.48	0.32	0.747	1.61	0.09	29.57
Bio-ADM 24h	0.05	0.03	1.72	0.086	1.05	0.99	1.10

Note. Estimates represent the log odds of "rehospitalization = 1" vs. "rehospitalization = 0"

Binomial Logistic Regression using Bio-ADM at 48h postoperatively and established risk-score to predict rehospitalization

Model Fit Measures

Model	Deviance	AIC	R^2_{McF}
1	18.42	28.42	0.32

Note. Models estimated using sample size of N=20

Model Coefficients - rehospitalization

Predictor	Estimate	SE	Z	p	Odds ratio	95% Confidence Interval	
						Lower	Upper
Intercept	-3.21	3.66	-0.88	0.380	0.04	0.00	52.54
EuroSCOREII	0.00	0.09	0.05	0.959	1.00	0.84	1.20
HeartMate3 risk score	0.45	0.89	0.51	0.613	1.57	0.27	9.08
the HMII risk score	-0.27	0.93	-0.29	0.769	0.76	0.12	4.69
Bio-ADM 48h	0.06	0.03	1.99	0.047	1.06	1.00	1.13

Note. Estimates represent the log odds of "rehospitalization = 1" vs. "rehospitalization = 0"

(OR: 1.06, 95%-CI: 1.00 – 1.13, p=0.047)

Prediction

Predictive Measures

Accuracy	Specificity	Sensitivity	AUC
0.80	0.92	0.63	0.74

Note. The cut-off value is set to 0.5

ROC Curve

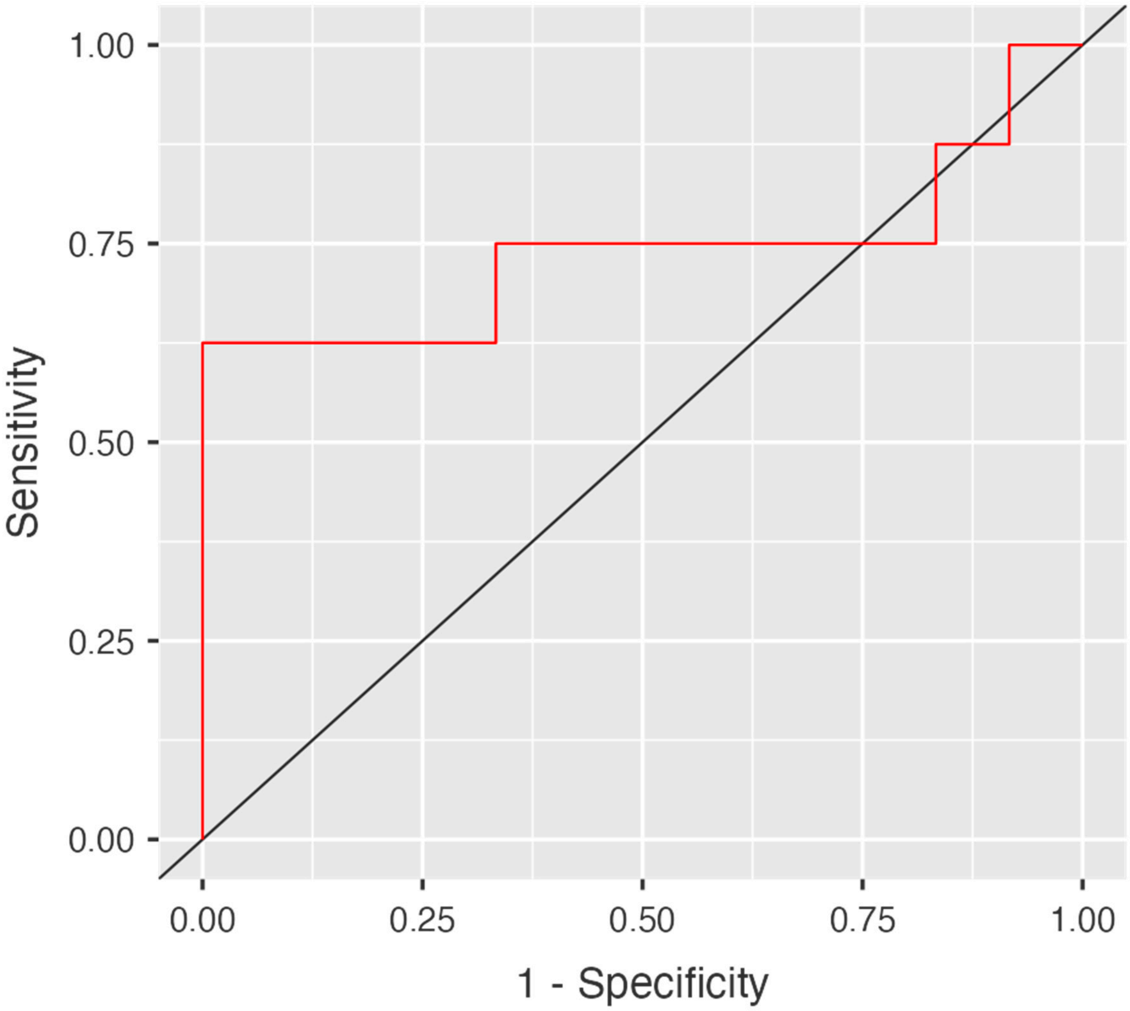
1.00
0.75
0.50
0.25
0.00
Sensitivity
0.00
0.25
0.50
0.75
1.00
1 - Specificity
