# Supplementary material for: Clinical Value of Bioactive Adrenomedullin and Proenkephalin A in Patients with Left Ventricular Assist Devices: An Observational Study
Source: J Clin Med. 2025 May 21;14(10):3613. doi: 10.3390/jcm14103613 (PMC12112301; doi:10.3390/jcm14103613)
Supplement: Supplementary file 1 [file jcm-14-03613-s001.zip › Supplementary File S2.pdf]

Supplementary File S2

Correlation analysis between penKid measurements at different time points and postoperative dialysis

Correlation Matrix

Correlation Matrix

Correlation Matrix

		postoperative_Dialysis	preop penKid	penKid at ICU	penKid 24h	penKid 48h
**postoperative_Dialysis**	**Spearman's rho**	—				
	**df**	—				
	**p-value**	—				
	**N**	—				
**preop penKid**	**Spearman's rho**	0.78***	—			
	**df**	18	—			
	**p-value**	<.001	—			
	**N**	20	—			
**penKid at ICU**	**Spearman's rho**	0.58**	0.79***	—		
	**df**	18	18	—		
	**p-value**	0.008	<.001	—		
	**N**	20	20	—		
**penKid 24h**	**Spearman's rho**	0.57**	0.68***	0.53*	—	
	**df**	18	18	18	—	
	**p-value**	0.009	<.001	0.017	—	
	**N**	20	20	20	—	
**penKid 48h**	**Spearman's rho**	0.27	0.14	0.12	0.58**	—
	**df**	18	18	18	18	—

**p-value**	0.241	0.544	0.61<br>0	0.007	—
**N**	20	20	20	20	—

Note. * p < .05, ** p < .01, *** p < .001

Binomial Logistic Regression for prediction of postoperative dialysis using penKid value at ICU admission and the kidney failure risk score

Model Fit Measures

Model	Deviance	AIC	R^2_{McF}
1	15.19	21.19	0.44

Note. Models estimated using sample size of N=20

Model Coefficients - postoperative_Dialysis

						95% Confidence Interval	
**Predictor**	**Estimate**	**SE**	**Z**	**p**	**Odds ratio**	**Lower**	**Upper**
Intercept	-3.59	1.41	-<br>2.55	0.011	0.03	0.00	0.44
pro_ENKserum_direct_postop	0.03	0.01	2.21	0.027	1.03	1.00	1.06
Kidney Failure Risk	0.08	0.18	0.45	0.654	1.08	0.76	1.55

Note. Estimates represent the log odds of "postoperative_Dialysis = 1" vs. "postoperative_Dialysis = 0"

(OR:1.08, 95%-CI: 0.76 – 1.55, *p*=0.027)

Prediction

Predictive Measures

Accuracy	Specificity	Sensitivity	AUC

0.85	0.92	0.75	0.82

Note. The cut-off value is set to 0.5

ROC Curve

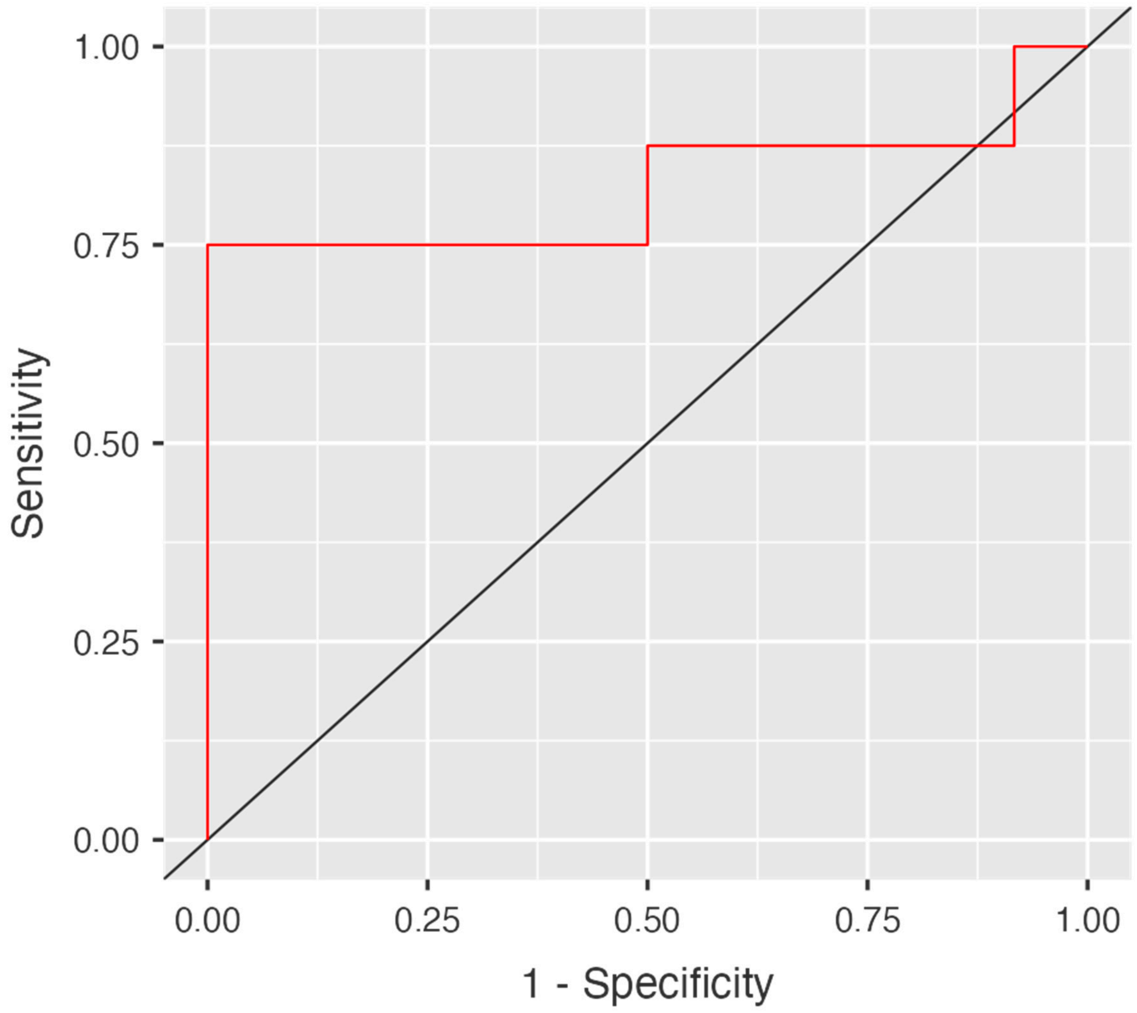

Binomial Logistic Regression for prediction of postoperative dialysis using penKid value at 24h postoperatively and the kidney failure risk score

Model Fit Measures

Model	Deviance	AIC	R^2_{McF}
1	21.96	27.96	0.18

Note. Models estimated using sample size of N=20

Model Coefficients - postoperative_Dialysis

Predictor	Estimate	SE	Z	p	Odds ratio	95% Confidence Interval	
						Lower	Upper
Intercept	-2.87	1.59	-1.80	0.072	0.06	0.00	1.30
penKid 24h	0.03	0.02	1.37	0.171	1.03	0.99	1.07
Kidney Failure Risk	0.19	0.20	0.95	0.341	1.21	0.82	1.79

Note. Estimates represent the log odds of "postoperative_Dialysis = 1" vs. "postoperative_Dialysis = 0"

Binomial Logistic Regression for prediction of postoperative dialysis using penKid value at 48h postoperatively and the kidney failure risk score

Model Fit Measures

Model	Deviance	AIC	R^2_{McF}
1	22.64	28.64	0.16

Note. Models estimated using sample size of N=20

Model Coefficients - postoperative_Dialysis

Predictor	Estimate	SE	Z	p	Odds ratio	95% Confidence Interval	
						Lower	Upper
Intercept	-2.08	1.21	-1.72	0.085	0.12	0.01	1.33
Kidney Failure Risk	0.18	0.18	1.04	0.297	1.20	0.85	1.69
penKid 48h	0.02	0.02	1.18	0.240	1.02	0.99	1.06

Note. Estimates represent the log odds of "postoperative_Dialysis = 1" vs. "postoperative_Dialysis = 0"

Correlation analysis between penKid measurements at different time points and 30-day mortality

Correlation Matrix

		30-days-mortality	preop penKid	penKid at ICU	penKid 24h	penKid 48h
**30-days-mortality**	**Spearman's rho**	—				
	**df**	—				

	p-value	—				
	N	—				
preop penKid	Spearman's rho	0.62**	—			
	df	18	—			
	p-value	0.004	—			
	N	20	—			
penKid at ICU	Spearman's rho	0.55*	0.79***	—		
	df	18	18	—		
	p-value	0.013	<.001	—		
	N	20	20	—		
penKid 24h	Spearman's rho	0.18	0.68***	0.53*	—	
	df	18	18	18	—	
	p-value	0.441	<.001	0.017	—	
	N	20	20	20	—	
penKid 48h	Spearman's rho	-0.01	0.14	0.12	0.58**	—
	df	18	18	18	18	—
	p-value	0.959	0.544	0.610	0.007	—
	N	20	20	20	20	—

Note. * p < .05, ** p < .01, *** p < .001

Binomial Logistic Regression using penKid value at ICU admission and established risk-score to predict 30-days mortality

Model Fit Measures

Model	Deviance	AIC	R^2_{McF}
1	0.00	10.00	1.00

Note. Models estimated using sample size of N=20

Model Coefficients - 30-days-mortality

Predictor	Estimate	SE	Z	p	Odds ratio	95% Confidence Interval Lower	Upper
Intercept	-231.12	477549.41	-0.00	1.000	0.00	0.00	Inf
penKid at ICU	0.68	1047.77	0.00	0.999	1.98	0.00	Inf
EuroSCORE II	-2.43	13799.37	-0.00	1.000	0.09	0.00	Inf
HeartMate 3 risk score	48.09	100864.32	0.00	1.000	765997794483021807616.00	0.00	Inf
the HMII risk score	-7.69	80246.58	-0.00	1.000	0.00	0.00	Inf

Note. Estimates represent the log odds of "30-days-mortality = 1" vs. "30-days-mortality = 0"

Binomial Logistic Regression using penKid value 24h postoperatively and established risk-score to predict 30-days mortality

Model Fit Measures

Model	Deviance	AIC	R^2_{McF}
1	15.69	25.69	0.07

Note. Models estimated using sample size of N=20

Model Coefficients - 30-days-mortality

Predictor	Estimate	SE	Z	p	Odds ratio	95% Confidence Interval Lower	Upper
Intercept	0.27	4.43	0.06	0.951	1.31	0.00	7731.70
EuroSCOREII	-0.05	0.14	-0.35	0.724	0.95	0.72	1.26
HeartMate3 risk score	-0.63	1.07	-0.59	0.557	0.54	0.07	4.32

the HMII risk score	-0.42	1.06	-0.40	0.692	0.66	0.08	5.27
penKid 24h	0.02	0.02	0.77	0.442	1.02	0.98	1.06

Note. Estimates represent the log odds of "30-days-mortality = 1" vs. "30-days-mortality = 0"

Binomial Logistic Regression using penKid value 48h postoperatively and established risk-score to predict 30-days mortality

Model Fit Measures

Model	Deviance	AIC	R^2_{McF}
1	15.30	25.30	0.10

Note. Models estimated using sample size of N=20

Model Coefficients - 30-days-mortality

						95% Confidence Interval	
Predictor	Estimate	SE	Z	p	Odds ratio	Lower	Upper
Intercept	0.77	4.45	0.17	0.862	2.17	0.00	13236.96
EuroSCOREII	-0.02	0.14	-0.18	0.854	0.98	0.75	1.27
HeartMate3 risk score	-0.72	1.08	-0.67	0.503	0.49	0.06	4.02
the HMII risk score	-0.64	1.12	-0.57	0.566	0.53	0.06	4.73
penKid 48h	0.02	0.02	0.99	0.322	1.02	0.98	1.05

Note. Estimates represent the log odds of "30-days-mortality = 1" vs. "30-days-mortality = 0"
