# Supplementary material for: Clinical Value of Bioactive Adrenomedullin and Proenkephalin A in Patients with Left Ventricular Assist Devices: An Observational Study
Source: J Clin Med. 2025 May 21;14(10):3613. doi: 10.3390/jcm14103613 (PMC12112301; doi:10.3390/jcm14103613)
Supplement: Supplementary file 1 [file jcm-14-03613-s001.zip › Table S1.pdf]

Table S1.
A: Multivariate logistic regression with post-LVAD RHF as a dependent variable

Coefficients

							Wald Test			95% Confidence interval (odds ratio scale)	
Model		Estimate	Robust Standard Error	Standardized⁺	Odds Ratio	z	Wald Statistic	df	p	Lower bound	Upper bound
M_0	(Intercept)	-2.415	1.459	-1.003	0.089	-1.655	5.829	1	0.098	0.005	1.560
	preadm	0.010	0.011	1.501	1.010	0.904	3.999	1	0.366	0.988	1.033
M_1	(Intercept)	-3.175	1.389	-1.437	0.042	-2.286	2.964	1	0.022	0.003	0.636
	preadm	0.014	0.008	2.006	1.014	1.639	4.293	1	0.043	0.997	1.031
	the Michigan RV score	-0.989	0.489	-1.609	0.372	-2.021	1.598	1	0.113	0.142	0.971
	The CRITT score	-0.381	0.865	-0.261	0.683	-0.440	0.173	1	0.660	0.125	3.722
	EUROMACS-RHF-Score	0.478	0.371	1.063	1.614	1.289	1.047	1	0.198	0.779	3.341

Note. postoop_RHF level '1' coded as class 1.

⁺ Standardized estimates represent estimates where the continuous predictors are standardized (X-standardization).

Bootstrap Coefficients

						95% bca* Confidence interval (odds ratio scale)	
	Estimate	Bias	Robust Standard Error	Standardized⁺	Odds Ratio	Lower bound	Upper bound
(Intercept)	-30.778	-121.858	1.514	-3.999	4.300×10^{-14}	0.000	∞
preadm	0.200	0.509	0.006	20.382	1.222	0.513	1.334
the Michigan RV score	-1.732	-52.588	0.656	-2.763	0.177	0.000	1191309133.094
The CRITT score	0.310	-26.352	1.039	0.189	1.363	0.000	44641592.370
EUROMACS-RHF-Score	0.443	43.000	0.429	0.936	1.558	0.002	1.8051342990367537e+29

* Bias corrected accelerated.

Note. Bootstrapping based on 4998 successful replicates.

Note. Coefficient estimate and robust standard error are based on the median of the bootstrap distribution.

⁺ Standardized estimates represent estimates where the continuous predictors are standardized (X-standardization).

B: Multivariate logistic regression with rehospitalization as a dependent variable

Coefficients

Model		Estimate	Robust Standard Error	Standardized$^+$	Odds Ratio	z	Wald Test			95% Confidence interval (odds ratio scale)	
							Wald Statistic	df	p	Lower bound	Upper bound
M_0	(Intercept)	-1.303	0.942	-0.408	0.272	-1.383	3.097	1	0.167	0.043	1.722
	preadm	0.007	0.008	0.951	1.007	0.830	2.431	1	0.407	0.991	1.022
M_1	(Intercept)	-1.056	3.494	-0.422	0.348	-0.302	0.127	1	0.763	0.000	328.033
	preadm	0.006	0.007	0.938	1.006	0.863	2.243	1	0.388	0.992	1.021
	HeartMate3 risk score	0.052	0.779	0.039	1.053	0.067	0.005	1	0.947	0.229	4.849
	the HMII risk score	-0.280	0.712	-0.199	0.755	-0.394	0.148	1	0.694	0.187	3.050
	EuroSCOREII	0.011	0.061	0.074	1.011	0.179	0.017	1	0.858	0.897	1.139

Note. rehospitalization level '1' coded as class 1.

$^+$ Standardized estimates represent estimates where the continuous predictors are standardized (X-standardization).
