# Supplementary material for: Clinical Value of Bioactive Adrenomedullin and Proenkephalin A in Patients with Left Ventricular Assist Devices: An Observational Study
Source: J Clin Med. 2025 May 21;14(10):3613. doi: 10.3390/jcm14103613 (PMC12112301; doi:10.3390/jcm14103613)
Supplement: Supplementary file 1 [file jcm-14-03613-s001.zip › Table S2.pdf]

## Table S2.

## A. Multivariate logistic regression with post-LVAD dialysis as a dependent variable

*Coefficients*

| | | | | | | | Wald Test | | | 95% Confidence interval (odds ratio scale) | |
|---|---|---|---|---|---|---|---|---|---|---|---|
| Model | | Estimate | Robust Standard Error | Standardized⁺ | Odds Ratio | z | Wald Statistic | df | p | Lower bound | Upper bound |
| $M_0$ | (Intercept) | -7.081 | 3.090 | -0.038 | $8.406 \times 10^{-4}$ | -2.291 | 4.785 | 1 | 0.022 | 0.000 | 0.359 |
| | preop penKid | 0.043 | 0.017 | 4.709 | 1.044 | 2.506 | 4.583 | 1 | 0.012 | 1.009 | 1.079 |
| $M_1$ | (Intercept) | -7.181 | 3.582 | -0.497 | $7.606 \times 10^{-4}$ | -2.005 | 4.328 | 1 | 0.045 | 0.000 | 0.852 |
| | preop penKid | 0.045 | 0.021 | 4.935 | 1.046 | 2.164 | 3.866 | 1 | 0.030 | 1.004 | 1.089 |
| | Kidney Failure Risk | -0.216 | 0.293 | -1.505 | 0.805 | -0.739 | 0.145 | 1 | 0.460 | 0.454 | 1.430 |

*Note.* postoperative_Dialysis level '1' coded as class 1.
⁺ Standardized estimates represent estimates where the continuous predictors are standardized (X-standardization).

*Bootstrap Coefficients*

| | | | | | | 95% bca* Confidence interval (odds ratio scale) | |
|---|---|---|---|---|---|---|---|
| | Estimate | Bias | Robust Standard Error | Standardized⁺ | Odds Ratio | Lower bound | Upper bound |
| (Intercept) | -8.728 | -28020671040858.777 | 3.765 | -0.768 | $1.620 \times 10^{-4}$ | 0.000 | 0.061 |
| preop penKid | 0.058 | 179985460208.306 | 0.023 | 6.033 | 1.059 | 1.006 | 4.746 |
| Kidney Failure Risk | -0.236 | -764555558520.429 | 0.338 | -1.401 | 0.789 | 0.000 | 2.5189097013739858e+44 |

* Bias corrected accelerated.
*Note.* Bootstrapping based on 5000 successful replicates.
*Note.* Coefficient estimate and robust standard error are based on the median of the bootstrap distribution.
⁺ Standardized estimates represent estimates where the continuous predictors are standardized (X-standardization).

## B. Multivariate logistic regression with 30-day mortality as a dependent variable

*Coefficients*

| | | | | | | | | Wald Test | | 95% Confidence interval (odds ratio scale) |
|---|---|---|---|---|---|---|---|---|---|---|
| Model | | Estimate | Robust Standard Error | Odds Ratio | z | Wald Statistic | df | p | Lower bound | Upper bound |
| $M_0$ | (Intercept) | -5.189 | 1.884 | 0.006 | -2.755 | 5.965 | 1 | 0.006 | 0.000 | 0.224 |
| | preop penKid | 0.016 | 0.010 | 1.016 | 1.639 | 4.249 | 1 | 0.101 | 0.997 | 1.035 |
| $M_1$ | (Intercept) | -3.568 | 4.685 | 0.028 | -0.762 | 0.249 | 1 | 0.446 | 0.000 | 274.055 |
| | preop penKid | 0.017 | 0.008 | 1.017 | 2.046 | 3.756 | 1 | 0.041 | 1.001 | 1.033 |
| | EuroSCOREII | -0.153 | 0.130 | 0.859 | -1.175 | 0.414 | 1 | 0.240 | 0.666 | 1.107 |
| | HeartMate3 risk score | 0.156 | 1.046 | 1.169 | 0.149 | 0.007 | 1 | 0.881 | 0.150 | 9.082 |
| | the HMII risk score | -0.303 | 0.834 | 0.739 | -0.363 | 0.052 | 1 | 0.716 | 0.144 | 3.784 |

*Note.* 30-days-mortality level '1' coded as class 1.

⁺ Standardized estimates represent estimates where the continuous predictors are standardized (X-standardization).

*Bootstrap Coefficients*

| | | | | | | 95% bca* Confidence interval (odds ratio scale) | |
|---|---|---|---|---|---|---|---|
| | Estimate | Bias | Robust Standard Error | Standardized⁺ | Odds Ratio | Lower bound | Upper bound |
| (Intercept) | -9.241 | -19564503996491.555 | 4.266 | -27.158 | $9.696 \times 10^{-5}$ | 0.000 | 8.376274170412766e+172 |
| preop penKid | 0.156 | -594311623.937 | 0.007 | 14.035 | 1.169 | 0.000 | 1.501 |

*Bootstrap Coefficients*

| | Estimate | Bias | Robust Standard Error | Standardized⁺ | Odds Ratio | 95% bca* Confidence interval (odds ratio scale) Lower bound | Upper bound |
|---|---|---|---|---|---|---|---|
| EuroSCOREII | -0.275 | 583196464732.948 | 0.117 | -1.806 | 0.759 | 0.002 | 6.180290593868004e+104 |
| HeartMate3 risk score | 0.159 | 1248837576082.669 | 1.006 | 0.114 | 1.173 | 0.000 | 1.1333842021761862e+56 |
| the HMII risk score | -0.028 | 3907175640722.975 | 0.878 | -0.019 | 0.972 | 0.000 | 172332837451629264896.000 |

* Bias corrected accelerated.

*Note.* Bootstrapping based on 5000 successful replicates.

*Note.* Coefficient estimate and robust standard error are based on the median of the bootstrap distribution.

⁺ Standardized estimates represent estimates where the continuous predictors are standardized (X-standardization).

## C: Multivariate logistic regression with sepsis as a dependent variable

*Coefficients*

| Model | | Estimate | Robust Standard Error | Standardized⁺ | Odds Ratio | z | Wald Test | | | 95% Confidence interval (odds ratio scale) | |
|---|---|---|---|---|---|---|---|---|---|---|---|
| | | | | | | | Wald Statistic | df | p | Lower bound | Upper bound |
| $M_0$ | (Intercept) | -5.189 | 1.884 | -2.598 | 0.006 | -2.755 | 5.965 | 1 | 0.006 | 0.000 | 0.224 |
| | preop penKid | 0.016 | 0.010 | 1.732 | 1.016 | 1.639 | 4.249 | 1 | 0.101 | 0.997 | 1.035 |
| $M_1$ | (Intercept) | -3.568 | 4.685 | -2.738 | 0.028 | -0.762 | 0.249 | 1 | 0.446 | 0.000 | 274.055 |
| | preop penKid | 0.017 | 0.008 | 1.833 | 1.017 | 2.046 | 3.756 | 1 | 0.041 | 1.001 | 1.033 |
| | EuroSCOREII | -0.153 | 0.130 | -1.035 | 0.859 | -1.175 | 0.414 | 1 | 0.240 | 0.666 | 1.107 |
| | HeartMate3 risk score | 0.156 | 1.046 | 0.119 | 1.169 | 0.149 | 0.007 | 1 | 0.881 | 0.150 | 9.082 |
| | the HMII risk score | -0.303 | 0.834 | -0.215 | 0.739 | -0.363 | 0.052 | 1 | 0.716 | 0.144 | 3.784 |

*Note.* postop Sepsis level '1' coded as class 1.

⁺ Standardized estimates represent estimates where the continuous predictors are standardized (X-standardization).

*Bootstrap Coefficients*

| | Estimate | Bias | Robust Standard Error | Standardized⁺ | Odds Ratio | 95% bca* Confidence interval (odds ratio scale) | |
|---|---|---|---|---|---|---|---|
| | | | | | | Lower bound | Upper bound |
| (Intercept) | -10.303 | -13257479501049.641 | 4.233 | -27.842 | $3.352\times10^{-5}$ | 0.000 | 3.2001620937627246e+170 |
| preop penKid | 0.154 | 899283046.969 | 0.007 | 14.708 | 1.167 | 0.598 | 1.847 |
| EuroSCOREII | -0.268 | 401773905371.647 | 0.116 | -1.728 | 0.765 | 0.002 | 1.0160520723210456e+106 |
| HeartMate3 risk score | 0.180 | 853128984382.154 | 1.002 | 0.133 | 1.197 | 0.000 | 4.08362461926767e+78 |
| the HMII risk score | -0.000 | 2657209477022.237 | 0.896 | -0.000 | 1.000 | 0.000 | 386153951668255522816.000 |

* Bias corrected accelerated.

*Note.* Bootstrapping based on 5000 successful replicates.

*Note.* Coefficient estimate and robust standard error are based on the median of the bootstrap distribution.

⁺ Standardized estimates represent estimates where the continuous predictors are standardized (X-standardization).
